# Immunotherapy and Metastatic Renal Cell Carcinoma: A Review of New Treatment Approaches

**DOI:** 10.3390/life12010024

**Published:** 2021-12-24

**Authors:** Nikhita Kathuria-Prakash, Claire Drolen, Christopher A. Hannigan, Alexandra Drakaki

**Affiliations:** 1 Department of Medicine, David Geffen School of Medicine, UCLA, Los Angeles, CA 90095, USA; nkathuria@mednet.ucla.edu (N.K.-P.); cdrolen@mednet.ucla.edu (C.D.); channigan@mednet.ucla.edu (C.A.H.); 2 Division of Hematology/Oncology, Department of Medicine, David Geffen School of Medicine, UCLA, Los Angeles, CA 90095, USA

**Keywords:** renal cell carcinoma, immunotherapy, immune checkpoint inhibitors, sunitinib, complete response rate

## Abstract

Introduction: Renal cell carcinomas (RCC) have been treated with immunotherapy for decades; the use of immune checkpoint inhibitors represents the most recent advance. In this review, we compare these new RCC immunotherapies, with a focus on achieving durable complete responses (CR). Review: Sorafenib and sunitinib were the first Food and Drug Administration (FDA)-approved targeted agents for RCC, with sunitinib eventually becoming the standard-of-care agent against which novel therapies are compared. In the last five years, many combination therapies based on the use of immune checkpoint inhibitors (ICIs) and receptor tyrosine kinase inhibitors (TKIs), including ipilimumab/nivolumab, nivolumab/cabozantinib, avelumab/axitinib, pembrolizumab/axitinib, and pembrolizumab/lenvatinib, have demonstrated superior overall survival (OS) and progression-free survival (PFS) compared to sunitinib. Ongoing clinical trials of hypoxia-induced factor-2 alpha (HIF-2a) inhibitors, chimeric antigen receptor T cell (CAR-T) therapy targeting CD70, and other new combination therapies have also shown promise and are currently under investigation. Conclusions: Many new combination therapies are approved for RCC treatment, and CR rates suggest that, in the era of immunotherapy, it may be possible to achieve durable responses and survival benefit in patients with metastatic RCC.

## 1. Introduction

Renal cell carcinomas (RCC) arise from the renal tubular epithelium and account for >80% of all cancers in the kidney. There are approximately 580,000 people living with RCC in the United States, with 76,000 new cases of RCC estimated in 2021, placing RCC among the top 10 most common cancers for both men and women [1]. Roughly 65% of people presenting with RCC have localized tumors, which can often be successfully managed with surgery. Those who develop disease recurrence after surgery, along with the remaining 35% who present initially with metastatic disease, require systemic therapy and have a poor prognosis, with an estimated five-year survival rate of 71.0% for patients with regional disease compared to only 13.9% for those with distant metastatic disease [1].

Immunotherapy treatments have long been utilized for the management of RCC. For decades, the cytokines interleukin 2 (IL-2) and interferon alpha were the only systemic treatments widely used for patients with unresectable RCC [2]. Durable complete responses (CR) are rarely achieved with the administration of high-dose IL-2; the overall response rates to this therapy are low (6–25%), and the treatments are associated with significant toxicity [2,3,4,5]. 

The past 20 years have seen significant progress in our understanding of the molecular drivers of RCC, which has, in turn, given rise to new treatment strategies. Research has primarily focused on the clear cell RCC (ccRCC) histology, which accounts for >75% of all cases [6]. Less common subtypes of RCC include papillary, chromophobe, medullary, collecting duct, sarcomatoid, and unclassified histology. Within ccRCC, the von Hippel Lindau (VHL) and hypoxia-induced factor (HIF) pathways have been identified as important drivers of pathogenesis. The loss of function of VHL promotes tumor angiogenesis via the unregulated expression of HIF1 alpha. Vascular endothelial growth factor (VEGF) receptors and the mechanistic target of rapamycin (mTOR) have been identified as actionable targets in the VHL/HIF axis, and multiple drugs targeting these molecules have been developed for the treatment of mRCC [7,8]. Perhaps the most notable among these is sunitinib, a first-in-class tyrosine kinase inhibitor of VEGF receptor, which demonstrated its superiority over interferon alpha in overall survival (OS), progression-free survival (PFS), and response rate (RR) in a landmark trial published in 2007 [9,10]. Sunitinib has since become the standard-of-care comparator for clinical trials in RCC. The results have been similar for trials with other VEGF receptor inhibitors, including sorafenib, pazopanib, axitinib, lenvatinib, cabozantinib, and bevacizumab [11,12,13,14,15,16,17,18]. The mTOR inhibitors temsirolimus and everolimus have also been shown to be clinically effective and are approved as largely second-line agents [19,20]. Unlike high-dose IL-2, the VEGF receptor and mTOR inhibitors typically do not yield durable clinical responses. The average PFS is on the order of 6-8 months for these agents when given as first- or second-line monotherapy [8,11,12,13,14,15,16,17,18].

Modern immune checkpoint inhibitors (ICI) targeting programmed cell death 1 (PD-1), programmed cell death ligand 1 (PD-L1), and cytotoxic T lymphocyte-associated antigen 4 (CTLA-4) have renewed the promise of immunotherapy in the treatment of RCC and show potential for achieving durable remission. PD-1 and CTLA-4 are both expressed on activated T cells and act to down-regulate T cell response when in contact with their ligands [21]. Cancer cells have leveraged this mechanism to evade immune surveillance by expressing PD-L1 [22]. Indeed, PD-L1 can be significantly overexpressed in RCC [22,23]. The treatments acting on this pathway include the PD-1 inhibitors nivolumab and pembrolizumab, the PD-L1 inhibitors avelumab and atezolizumab, and the CTLA-4 inhibitor ipilimumab.

In this review, we will discuss these current approaches to the treatment of RCC, focusing specifically on ICI/tyrosine kinase inhibitor (TKI) combinations (Table 1). Our aim is to describe, evaluate, and compare survival outcomes, with a focus on the rate of CR for the treatment of patients with metastatic RCC.

## 2. Current Therapies

### 2.1. Sunitinib

Sunitinib is an oral TKI that acts on VEGF receptors. It was granted accelerated approval by the FDA in 2006, based on two single-arm trials in patients with mRCC. Sunitinib demonstrated partial responses (PR) in 25.5% and 36.5% of patients [25,26]. In 2007, a randomized, multicenter phase III trial demonstrated the superiority of sunitinib over interferon alfa [9]. The sunitinib group had a longer median PFS and higher RR than the interferon alfa group (11 months vs. 5 months), as well as better quality of life [9]. With these results, sunitinib replaced interferon alpha as the standard-of-care therapy in RCC.

In a later study, pooled data were used to identify which patients would benefit most from sunitinib. Predictors of long-term OS (≥30 months) included ethnic origin (50.2 vs. 38.4 months in white vs. nonwhite patients), bone metastases (42.7 vs. 54.5 months for patients with vs. without metastases), and corrected calcium (41.7 vs. 50.2 months for patients with >10 vs. ≤10 mg/dL calcium) [27]. More recently, sunitinib was approved as an adjuvant treatment for patients with locoregional, high-risk ccRCC after a nephrectomy and demonstrated a longer median duration of disease-free survival than the placebo (6.8 years vs. 5.6 years) [28]. Overall, sunitinib was the first agent in the new era of targeted therapies to improve survival for patients with mRCC compared to prior cytokine-based treatments. In August of 2021, pembrolizumab was granted priority review for the adjuvant treatment of patients with RCC at intermediate or high risk of recurrence following a nephrectomy.

### 2.2. Ipilimumab & Nivolumab

Ipilimumab, a CTLA-4 inhibitor, and nivolumab, a PD-1 inhibitor, were the next therapies to be studied in mRCC, and the first and only dual ICI combination therapy to be approved for use in mRCC. The randomized, phase III CheckMate 214 trial assigned patients to receive either a combination of ipilimumab and nivolumab or sunitinib, and the primary outcome was 18-month OS in the patients with intermediate- or poor-risk groups by the International Metastatic RCC Database Consortium (IMDC) [24]. The 18-month OS rate was 75% with nivolumab and ipilimumab as compared to 60% with sunitinib, and the CR rate was 9% compared to 1%. This landmark trial established ipilimumab and nivolumab combination therapy as the first ICIs to demonstrate a durable response to therapy [24].

In 2020, the extended four-year follow-up data from the CheckMate 214 trial were published [29]. OS remained superior for the nivolumab and ipilimumab group compared to the sunitinib group, and the four-year PFS probability for the nivolumab and ipilimumab group was 31.0% compared to 17.3% in the sunitinib group [29]. Additionally, the probability of response for greater than four years was higher with nivolumab and ipilimumab than with sunitinib (59% vs. 30%), again suggesting the durability of response with combination ICI therapy. These data suggest that the combination therapy with nivolumab and ipilimumab offers a potentially long-lasting response for patients with mRCC, although more long-term data are needed to further quantify this.

### 2.3. Atezolizumab & Bevacizumab

Bevacizumab is an antibody that inhibits the VEGF receptor and was investigated in combination with atezolizumab, an anti-PD-L1 antibody, for patients with ccRCC or sarcomatoid histology RCC in the IMmotion151 phase III trial [13]. The trial enrolled 915 patients for a minimum of 12 months of follow-up. The median PFS for the patients receiving atezolizumab and bevacizumab (11.2 months) was significantly longer than for the patients receiving sunitinib (8.4 months) [13]. In the atezolizumab and bevacizumab group, 37% of patients achieved an objective response (OR), and 5% achieved a CR, whereas 33% of patients in the sunitinib group achieved an OR, and 2% achieved a CR, neither of which reached significance [13]. However, at a longer follow-up of 40 months, there was no survival benefit with atezolizumab and bevacizumab compared to sunitinib [30]. While this combination did not show a survival benefit or statistically significant CR rate, we are still treating three patients at our institution for over seven years now as part of this trial, which suggests again that this combination can produce durable responses in a subset of patients, even if it has not received FDA approval. Interestingly, a similar phase II trial is in progress for the treatment of naïve patients with mRCC looking at atezolizumab and bevacizumab in combination with a selective inhibitor of phosphoinositide-3-kinase (PI3K)–gamma (IPI-549) [31]. The study has completed accrual; however, no data are available at the time of this manuscript [31].

### 2.4. Axitinib & Avelumab

The next combination therapy investigated was axitinib, a highly selective VEGF receptor TKI, and avelumab, an anti-PD-L1 antibody. This combination was initially studied in a phase Ib trial that established its safety as combination therapy and suggested antitumor activity in patients with advanced ccRCC [32]. The phase III clinical trial, JAVELIN Renal 101, randomized 873 patients to receive either axitinib and avelumab or sunitinib [14]. The initial data were published in March 2019. Overall, the patients in the axitinib and avelumab group had significantly longer PFS (13.8 months) than those in the sunitinib group (8.4 months) [14]. The subgroup of patients with PD-L1-positive tumors also had significantly longer PFS after treatment with axitinib and avelumab (13.8 months) than the patients with PD-L1-positive tumors who received sunitinib (7.2 months) [14]. This subgroup also had significantly higher OR rates (55.2%), defined as a PR or CR, than the patients who received sunitinib (25.5%) [14]. However, confirmed CR rates were only 4.4% in the axitinib and avelumab group compared to 2.1% in the sunitinib group [14].

Updated data were published in August 2020, and again demonstrated significantly longer PFS in the axitinib and avelumab group than in the sunitinib group, as well as similar overall RR. However, CR rates in the combination group were only 3.8% compared to 2.0% in the sunitinib group [33]. Additional follow-up is still ongoing. The combination of axitinib and avelumab is a superior option to sunitinib for PFS but a durable CR has yet to be demonstrated. This study raises the question of whether the combination of targeted VEGF receptor inhibitor therapy and ICI have additive or synergistic effects.

### 2.5. Axitinib & Pembrolizumab

A similar phase III trial comparing sunitinib to another VEGF receptor TKI and anti-PD-1 regimen was published at the same time as JAVELIN Renal 101. The KEYNOTE-426 trial randomly assigned 861 patients with advanced ccRCC to receive axitinib and pembrolizumab or sunitinib [34]. Initial follow-up data at 12.8 months demonstrated significantly longer PFS in the axitinib and pembrolizumab group (15.1 months) than in the sunitinib group (11.1 months) [34]. The OR rate was also significantly higher in the pembrolizumab and axitinib group (59.3%) than in the sunitinib group (35.7%), and these findings were noted across IMDC risk groups and PD-L1 expression groups [34]. In the axitinib and pembrolizumab group, 5.8% of patients achieved a CR, compared to 1.9% of patients in the sunitinib group [34].

The first interim analysis was published 15 months later, reporting a median follow-up time of 30.6 months [35]. PFS was significantly longer in the axitinib and pembrolizumab group (15.4 months) than in the sunitinib group (11.1 months) [35]. Additionally, the OR rate was higher in the axitinib and pembrolizumab group (60%) than in the sunitinib group (40%), and 9% of patients in the axitinib and pembrolizumab group achieved a CR compared to 3% in the sunitinib group [35]. The second interim analysis data were presented at the American Society of Clinical Oncology Annual Meeting in June 2021, with a 42.8-month median follow-up, the longest follow-up of any ICI and TKI combination therapy [36]. Again, patients who received pembrolizumab and axitinib had significantly longer PFS (15.7 months) and a significantly higher rate of PFS (25.1%) than those who received sunitinib (11 months, 10.6%) [36]. The OR rate was also higher for patients who received pembrolizumab and axitinib (60.4%) than for patients who received sunitinib (39.6%) [36]. The CR rate was higher in the pembrolizumab and axitinib group (10.0%) than in the sunitinib group (3.5%), but the trend did not achieve statistical significance [36]. This was the final analysis of the KEYNOTE-426 trial and demonstrates the improved PFS and OR rates with axitinib and pembrolizumab [36].

### 2.6. Cabozantinib & Nivolumab

The CheckMate 9ER trial studied nivolumab, an anti-PD-1 antibody, and cabozantinib, a tyrosine kinase inhibitor that regulates tumor cell proliferation, neovascularization, and immune cell regulation through the inhibition of VEGF, cMET, and other receptor-based targets [16]. The phase III clinical trial enrolled 651 patients with previously untreated advanced ccRCC, defined as either unamenable to curative surgery or radiation or metastatic disease [16]. At a median follow-up of 18.1 months, the median PFS was significantly longer for the patients in the cabozantinib and nivolumab group (16.6 months) than for the patients in the sunitinib group (8.3 months) [16]. Significantly more patients had an OR with cabozantinib and nivolumab (55.7%) compared to with sunitinib (27.1%) [16]. In the cabozantinib and nivolumab group, 8.0% of patients achieved a CR, compared to 4.6% of patients in the sunitinib group [16]. These findings persisted across IMDC risk subgroups and tumor PD-L1 expression subgroups [16]. Updated analyses from extended follow-up have not yet been published. Based on these findings, the combination of nivolumab and cabozantinib is now one of the first-line combination ICI and targeted therapies recommended for treatment-naïve mRCC [37]. Although the available data suggest a trend towards a CR with combination therapy, statistical significance has not yet been established. However, there is hope that this trend may be established with an extended follow-up of the CheckMate 9ER trial, especially as more patients now have the option to be treated with this combination given its recent FDA approval [38].

### 2.7. Lenvatinib & Pembrolizumab

The most recent phase III combination trial to be published was the three-armed CLEAR trial. This trial studied lenvatinib, an antiangiogenic agent, and pembrolizumab, an anti-PD-1 antibody, or lenvatinib and everolimus, an mTOR kinase inhibitor, versus sunitinib [39]. A total of 1069 patients were randomized for a median of 17 months of follow-up and found that PFS was significantly longer with lenvatinib and pembrolizumab (23.9 months) than with sunitinib (9.2 months) [39]. The proportion of patients with an OR was significantly higher in the lenvatinib and pembrolizumab group (71.0%) than in the sunitinib group (36.1%), and 16.1% of patients in the lenvatinib and pembrolizumab group achieved a CR compared to 4.2% of patients in the sunitinib group [39]. Longer-term follow-up data are being collected, and the study is set to end 31 July 2022 [40]. The initial CR data are promising for the lenvatinib and pembrolizumab group, since this is the highest reported CR rate of the studies reviewed, although the follow-up time is shorter than the data reported for other combination ICI and targeted therapies. Longer follow-up data may establish lenvatinib and pembrolizumab as a superior combination for achieving a CR.

## 3. Future Therapies (Ongoing Clinical Trials)

The rise of ICIs for metastatic RCC has also increased the need for alternative therapeutic strategies for patients who do not respond or become refractory to currently available treatment. Many ongoing clinical trials aim to provide those options. These include novel agents, as well as new combinations of previously approved agents, some of which were discussed above. Each of the trials discussed below are actively recruiting subjects at the time of this publication; more information, including the available study locations and enrollment criteria, can be found on ClinicalTrials.gov, accessed on 12 September 2021.

### 3.1. HIF-2 Alpha Inhibitors

**Figure 1:**
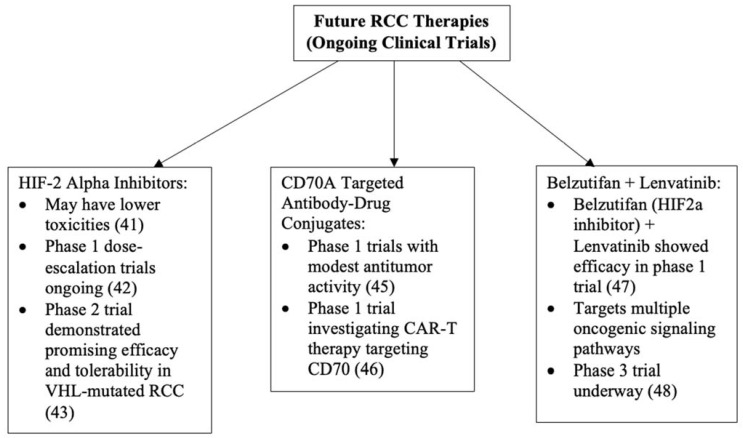
Directions for future RCC therapies [41,42,43,45,46,47,48].

**Figure 2:**
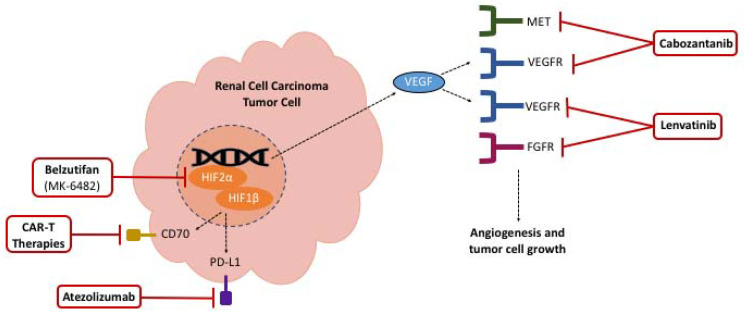
Mechanisms of future RCC therapies.

Novel agents such as HIF-2 alpha inhibitors and anti-CD70A therapies are being evaluated as potential treatments for ccRCC. HIF-2 alpha inhibitors are orally active, and initial studies suggest that these agents may have lower toxicity than sunitinib (Figure 1 and Figure 2) [41]. Phase I dose-escalation trials with HIF-2 alpha inhibitors in combination with nivolumab or cabozantinib are ongoing in patients with ccRCC, with planned completion dates in November 2022 [42]. A recent phase II trial with the HIF-2 alpha inhibitor belzutifan (MK-6482) demonstrated promising efficacy and tolerability in patients with VHL-mutated cancers, including RCC, and the drug has recently been approved by the FDA for this indication [43,44].

### 3.2. CD70A Targeted Antibody-Drug Conjugates

Meanwhile, a single-agent antibody-drug conjugate targeting CD70 is being evaluated to treat patients with CD70-positive RCC [45]. The results of a phase I clinical trial reported only modest antitumor activity, but anti-CD70 therapies may still be a desirable treatment option for patients with advanced, refractory RCC [45]. At the time of this manuscript, a phase I trial is underway to investigate the safety and efficacy of an allogeneic chimeric antigen receptor T cell (CAR-T) therapy targeting CD70 (Figure 1 and Figure 2) [46]. The study started in February 2021 and aims to complete enrollment of 48 patients by December 2022. Given the success rates of CAR therapies in hematologic malignancies, and other cell therapies for both hematologic and solid malignancies, it is hoped that this will be the next generation of therapies to advance cure rates for RCC [47].

### 3.3. MK-6482-011

The Merck phase III MK-6482-011 study is evaluating the safety and efficacy of belzutifan and lenvatinib compared with cabozantinib as a second- or third-line therapy after progression on a PD-1/L1 agent in patients with advanced ccRCC [48,49,50]. As discussed above, belzutifan is a highly selective oral HIF-2 alpha inhibitor approved for use in VHL-mutated RCC and is now being investigated in combination with lenvatinib for patients with advanced RCC [43]. In the preliminary phase III clinical study, belzutifan and lenvatinib showed efficacy among a 55-subject RCC cohort, with 13 (24%) patients achieving a PR, and 31 (56%) developing stable disease [48]. The combination of belzutifan and lenvatinib is thought to inhibit the VEGF and multiple other oncogenic signaling pathways responsible for progression and metastasis (Figure 1 and Figure 2). The subjects must have advanced ccRCC, measurable disease by imaging criteria, available tissue for central analysis, and good performance status [49]. Subject recruitment began in February 2021, and the study is expected to enroll 708 participants by 2024 [49].

### 3.4. CONTACT-03

The F. Hoffmann-La Roche phase III WO41994 study (CONTACT-03) is an ongoing clinical trial comparing the efficacy and safety of atezolizumab and cabozantinib to cabozantinib monotherapy in patients with locally advanced or metastatic RCC following a progression on ICI therapy (Figure 1 and Figure 2) [50]. The cohorts are open for ccRCC and non-ccRCC, including papillary, chromophobe and unclassified subtypes [50]. Enrollment is dependent on radiologic progression on or within 6 months after the last dose of anti-PD-1/L1 therapy, no previous mTOR therapy, measurable disease by imaging criteria, available tissue for central analysis, and good performance status [50]. Both study drugs have been approved in combination with other agents, as discussed above, and have been studied together in the phase I COSMIC-021 trial for patients with previously untreated disease across multiple solid tumor types, including ccRCC and non-ccRCC [51]. It is hypothesized that cabozantinib enhances the antitumor effect of anti-PD-L1 agents by increasing T cell tumor infiltration [52]. Enrollment for CONTACT-03 is not yet complete, but an interim analysis showed efficacy in both RCC cohorts, with one CR, four confirmed PRs, and two unconfirmed PRs across 10 patients [52]. Enrollment opened in July 2020, and the trial is expected to enroll 500 participants by 2022 [51].

## 4. Conclusions

In conclusion, the treatment options for mRCC have expanded markedly since the discovery and approval of IL-2 in the 1990s. Starting with the VEGF receptor TKI sunitinib, and the more recently approved ICI/TKI combination therapies, providers now have multiple options for a first-line immunotherapy for patients with mRCC. While none of these combination therapies have achieved statistically significant CR thus far, the data are promising. Additional therapies are in the pipeline, including HIF-2a inhibitors, antibody-drug conjugates and CARs targeting CD70, as well as many ongoing studies with combination therapies, and these therapies may again change the field of mRCC. With a longer follow-up and larger sample sizes, we may be entering a new era of “curing” patients with mRCC.

## Figures and Tables

**Table 1:** RCC Trials by Clinical Phase.

Trial	Therapies Evaluated	Clinical Trial Phase
Motzer et al., 2007 [9]	sunitinib vs. interferon alfa	Phase 3
Motzer et al., 2018 (CheckMate 214) [24]	ipilimumab + nivolumab vs. sunitinib	Phase 3
Rini et al., 2019 (IMmotion 151) [13]	bevacizumab + atezolizumab vs. sunitinib	Phase 3
Motzer et al., 2019 (JAVELIN Renal 101) [14]	axitinib + avelumab vs. sunitinib	Phase 3
Rini et al., 2019 (KEYNOTE-426) [13]	axitinib + pembrolizumab vs. sunitinib	Phase 3
Choueiri et al., 2021 (CheckMate 9ER) [16]	nivolumab + cabozantinib vs. sunitinib	Phase 3
Motzer et al., 2021 (CLEAR) [15]	lenvatinib + pembrolizumab vs. lenvatinib + everolimus vs. sunitinib	Phase 3

## Data Availability

Not applicable.

## References

[1] Cancer of the Kidney and Renal Pelvis—Cancer Stat Facts SEER. https://seer.cancer.gov/statfacts/html/kidrp.html.

[2] Negrier S., Escudier B., Lasset C., Douillard J.-Y., Savary J., Chevreau C., Ravaud A., Mercatello A., Peny J., Mousseau M. (1998). Recombinant Human Interleukin-2, Recombinant Human Interferon Alfa-2a, or Both in Metastatic Renal-Cell Carcinoma. N. Engl. J. Med..

[3] Fyfe G., Fisher R.I., Rosenberg S.A., Sznol M., Parkinson D.R., Louie A.C. (1995). Results of treatment of 255 patients with metastatic renal cell carcinoma who received high-dose recombinant interleukin-2 therapy. J. Clin. Oncol..

[4] McDermott D.F., Regan M.M., Clark J.I., Flaherty L.E., Weiss G.R., Logan T.F., Kirkwood J.M., Gordon M.S., Sosman J.A., Ernstoff M.S. (2005). Randomized Phase III Trial of High-Dose Interleukin-2 Versus Subcutaneous Interleukin-2 and Interferon in Patients with Metastatic Renal Cell Carcinoma. J. Clin. Oncol..

[5] McDermott D.F., Cheng S.-C., Signoretti S., Margolin K., Clark J.I., Sosman J.A., Dutcher J.P., Logan T.F., Curti B.D., Ernstoff M.S. (2015). The High-Dose Aldesleukin “Select” Trial: A Trial to Prospectively Validate Predictive Models of Response to Treatment in Patients with Metastatic Renal Cell Carcinoma. Clin. Cancer Res..

[6] Patard J.-J., Leray E., Rioux-Leclercq N., Cindolo L., Ficarra V., Zisman A., De La Taille A., Tostain J., Artibani W., Abbou C.C. (2005). Prognostic Value of Histologic Subtypes in Renal Cell Carcinoma: A Multicenter Experience. J. Clin. Oncol..

[7] Lainakis G., Bamias A. (2013). Targeting angiogenesis in renal cell carcinoma. Expert Opin. Pharmacother..

[8] Posadas E.M., Limvorasak S., Figlin R.A. (2017). Targeted therapies for renal cell carcinoma. Nat. Rev. Nephrol..

[9] Motzer R.J., Hutson T.E., Tomczak P., Michaelson M.D., Bukowski R.M., Rixe O., Oudard S., Negrier S., Szczylik C., Kim S.T. (2007). Sunitinib versus Interferon Alfa in Metastatic Renal-Cell Carcinoma. N. Engl. J. Med..

[10] Chow L.Q., Eckhardt S.G. (2007). Sunitinib: From Rational Design to Clinical Efficacy. J. Clin. Oncol..

[11] Escudier B., Eisen T., Stadler W.M., Szczylik C., Oudard S., Siebels M., Negrier S., Chevreau C., Solska E., Desai A.A. (2007). Sorafenib in Advanced Clear-Cell Renal-Cell Carcinoma. N. Engl. J. Med..

[12] Sternberg C.N., Davis I.D., Mardiak J., Szczylik C., Lee E., Wagstaff J., Barrios C.H., Salman P., Gladkov O.A., Kavina A. (2010). Pazopanib in Locally Advanced or Metastatic Renal Cell Carcinoma: Results of a Randomized Phase III Trial. J. Clin. Oncol..

[13] Rini B.I., Powles T., Atkins M.B., Escudier B., McDermott D.F., Suarez C., Bracarda S., Stadler W.M., Donskov F., Lee J.L. (2019). Atezolizumab plus bevacizumab versus sunitinib in patients with previously untreated metastatic renal cell carcinoma (IMmotion151): A multicentre, open-label, phase 3, randomised controlled trial. Lancet.

[14] Motzer R.J., Penkov K., Haanen J., Rini B., Albiges L., Campbell M.T., Venugopal B., Kollmannsberger C., Negrier S., Uemura M. (2019). Avelumab plus Axitinib versus Sunitinib for Advanced Renal-Cell Carcinoma. N. Engl. J. Med..

[15] Motzer R.J., Hutson T.E., Glen H., Michaelson D., Molina A., Eisen T., Jassem J., Zolnierek J., Maroto J.P., Mellado B. (2015). Lenvatinib, everolimus, and the combination in patients with metastatic renal cell carcinoma: A randomised, phase 2, open-label, multicentre trial. Lancet Oncol..

[16] Choueiri T.K., Powles T., Burotto M., Escudier B., Bourlon M.T., Zurawski B., Oyervides Juárez V.M., Hsieh J.J., Basso U., Shah A.Y. (2021). Nivolumab plus Cabozantinib versus Sunitinib for Advanced Renal-Cell Carcinoma. N. Engl. J. Med..

[17] Choueiri T.K., Escudier B., Powles T., Mainwaring P.N., Rini B.I., Donskov F., Hammers H., Hutson T.E., Lee J.-L., Peltola K. (2015). Cabozantinib versus Everolimus in Advanced Renal-Cell Carcinoma. N. Engl. J. Med..

[18] Rini B.I., Halabi S., Rosenberg J.E., Stadler W.M., Vaena D.A., Ou S.-S., Archer L., Atkins J.N., Picus J., Czaykowski P. (2008). Bevacizumab Plus Interferon Alfa Compared with Interferon Alfa Monotherapy in Patients with Metastatic Renal Cell Carcinoma: CALGB 90206. J. Clin. Oncol..

[19] Hudes G., Carducci M., Tomczak P., Dutcher J., Figlin R., Kapoor A., Staroslawska E., Sosman J., McDermott D., Bodrogi I. (2007). Temsirolimus, Interferon Alfa, or Both for Advanced Renal-Cell Carcinoma. N. Engl. J. Med..

[20] Motzer R.J., Escudier B., Oudard S., Do T.E.H., Porta C., Bracarda S., Grünwald V., Thompson J.A., Figlin R.A., Hollaender N. (2010). Phase 3 trial of everolimus for metastatic renal cell carcinoma: Final results and analysis of prognostic factors. Cancer.

[21] Pardoll D.M. (2012). The blockade of immune checkpoints in cancer immunotherapy. Nat. Rev. Cancer.

[22] Wang X., Lopez R., Luchtel R.A., Hafizi S., Gartrell B., Shenoy N. (2021). Immune evasion in renal cell carcinoma: Biology, clinical translation, future directions. Kidney Int..

[23] Thompson R.H., Dong H., Kwon E.D. (2007). Implications of B7-H1 Expression in Clear Cell Carcinoma of the Kidney for Prognostication and Therapy. Clin. Cancer Res..

[24] Motzer R.J., Tannir N.M., McDermott D.F., Arén Frontera O., Melichar B., Choueiri T.K., Plimack E.R., Barthélémy P., Porta C., George S. (2018). Nivolumab plus Ipilimumab versus Sunitinib in Advanced Renal-Cell Carcinoma. N. Engl. J. Med..

[25] Motzer R.J., Rini B.I., Bukowski R.M., Curti B.D., George D.J., Hudes G.R., Redman B.G., Margolin K., Merchan J.R., Wilding G. (2006). Sunitinib in Patients with Metastatic Renal Cell Carcinoma. JAMA.

[26] Motzer R.J., Michaelson M.D., Redman B.G., Hudes G.R., Wilding G., Figlin R.A., Ginsberg M.S., Kim S.T., Baum C.M., DePrimo S.E. (2006). Activity of SU11248, a Multitargeted Inhibitor of Vascular Endothelial Growth Factor Receptor and Platelet-Derived Growth Factor Receptor, in Patients with Metastatic Renal Cell Carcinoma. J. Clin. Oncol..

[27] Motzer R.J., Escudier B., Bukowski R., Rini B.I., Hutson T.E., Barrios C.H., Lin X., Fly K., Matczak E., Gore M.E. (2013). Prognostic factors for survival in 1059 patients treated with sunitinib for metastatic renal cell carcinoma. Br. J. Cancer.

[28] Ravaud A., Motzer R.J., Pandha H.S., George D.J., Pantuck A.J., Patel A., Chang Y.-H., Escudier B., Donskov F., Magheli A. (2016). Adjuvant Sunitinib in High-Risk Renal-Cell Carcinoma after Nephrectomy. N. Engl. J. Med..

[29] Albiges L., Tannir N.M., Burotto M., McDermott D., Plimack E.R., Barthélémy P., Porta C., Powles T., Donskov F., George S. (2020). Nivolumab plus ipilimumab versus sunitinib for first-line treatment of advanced renal cell carcinoma: Extended 4-year follow-up of the phase III CheckMate 214 trial. ESMO Open.

[30] Rini B.I., Atkins M.B., Escudier B., Powles T., McDermott D.F., Alekseev B.Y., Lee J.-L., Stroyakovskiy D., Rodriguez C.S., De Giorgi U. (2021). Abstract CT188: IMmotion 151: Updated overall survival (OS) and exploratory analysis of the association of gene expression and clinical outcomes with atezolizumab plus bevacizumab vs sunitinib in patients with locally advanced or metastatic renal cell carcinoma (mRCC). Clin. Trials.

[31] Infinity Pharmaceuticals, Inc (2020). A Phase 2, Multi-Arm, Multicenter, Open-Label Study to Evaluate the Efficacy and Safety of IPI-549 Administered in Combination with Front-Line Treatment Regimens in Patients with Locally Advanced and/or Metastatic Triple-Negative Breast Cancer or Renal Cell Carcinoma. https://clinicaltrials.gov/ct2/show/NCT03961698.

[32] Choueiri T.K., Larkin J., Oya M., Thistlethwaite F., Martignoni M., Nathan P., Powles T., McDermott D., Robbins P.B., Chism D.D. (2018). Preliminary results for avelumab plus axitinib as first-line therapy in patients with advanced clear-cell renal-cell carcinoma (JAVELIN Renal 100): An open-label, dose-finding and dose-expansion, phase 1b trial. Lancet Oncol..

[33] Choueiri T., Motzer R., Rini B., Haanen J., Campbell M., Venugopal B., Kollmannsberger C., Gravis-Mescam G., Uemura M., Lee J. (2020). Updated efficacy results from the JAVELIN Renal 101 trial: First-line avelumab plus axitinib versus sunitinib in patients with advanced renal cell carcinoma. Ann. Oncol..

[34] Rini B.I., Plimack E.R., Stus V., Gafanov R., Hawkins R., Nosov D., Pouliot F., Alekseev B., Soulières D., Melichar B. (2019). Pembrolizumab plus Axitinib versus Sunitinib for Advanced Renal-Cell Carcinoma. N. Engl. J. Med..

[35] Powles T., Plimack E.R., Soulières D., Waddell T., Stus V., Gafanov R., Nosov D., Pouliot F., Melichar B., Vynnychenko I. (2020). Pembrolizumab plus axitinib versus sunitinib monotherapy as first-line treatment of advanced renal cell carcinoma (KEYNOTE-426): Extended follow-up from a randomised, open-label, phase 3 trial. Lancet Oncol..

[36] Rini B.I., Plimack E.R., Stus V., Waddell T., Gafanov R., Pouliot F., Nosov D., Melichar B., Soulieres D., Borchiellini D. (2021). Pembrolizumab (pembro) plus axitinib (axi) versus sunitinib as first-line therapy for advanced clear cell renal cell carcinoma (ccRCC): Results from 42-month follow-up of KEYNOTE-426. J. Clin. Oncol..

[37] Bedke J., Albiges L., Capitanio U., Giles R.H., Hora M., Lam T.B., Ljungberg B., Marconi L., Klatte T., Volpe A. (2021). Updated European Association of Urology Guidelines on Renal Cell Carcinoma: Nivolumab plus Cabozantinib Joins Immune Checkpoint Inhibition Combination Therapies for Treatment-naïve Metastatic Clear-Cell Renal Cell Carcinoma. Eur. Urol..

[38] FDA Research C for DE and FDA Approves Nivolumab Plus Cabozantinib for Advanced Renal Cell Carcinoma. https://www.fda.gov/drugs/resources-information-approved-drugs/fda-approves-nivolumab-plus-cabozantinib-advanced-renal-cell-carcinoma.

[39] Motzer R., Alekseev B., Rha S.-Y., Porta C., Eto M., Powles T., Grünwald V., Hutson T.E., Kopyltsov E., Méndez-Vidal M.J. (2021). Lenvatinib plus Pembrolizumab or Everolimus for Advanced Renal Cell Carcinoma. N. Engl. J. Med..

[40] Eisai Inc (2021). A Multicenter, Open-Label, Randomized, Phase 3 Trial to Compare the Efficacy and Safety of Lenvatinib in Combination with Everolimus or Pembrolizumab Versus Sunitinib Alone in First-Line Treatment of Subjects with Advanced Renal Cell Carcinoma (CLEAR). https://clinicaltrials.gov/ct2/show/NCT02811861.

[41] Cuvillier O. (2017). The therapeutic potential of HIF-2 antagonism in renal cell carcinoma. Transl. Androl. Urol..

[42] Peloton Therapeutics, Inc (2021). A Phase 1, Multiple-Dose, Dose-Escalation Trial of PT2385 Tablets, a HIF-2α Inhibitor, in Patients with Advanced Clear Cell Renal Cell Carcinoma. https://clinicaltrials.gov/ct2/show/NCT02293980.

[43] FDA Research C for DE and FDA Approves Belzutifan for Cancers Associated with von Hippel-Lindau Disease. https://www.fda.gov/drugs/resources-information-approved-drugs/fda-approves-belzutifan-cancers-associated-von-hippel-lindau-disease.

[44] Jonasch E., Donskov F., Iliopoulos O., Rathmell W.K., Narayan V., Maughan B.L., Oudard S., Else T., Maranchie J.K., Welsh S.J. (2020). Phase II study of the oral HIF-2α inhibitor MK-6482 for Von Hippel-Lindau disease–associated renal cell carcinoma. J. Clin. Oncol..

[45] Pal S.K., Forero-Torres A., Thompson J.A., Morris J.C., Chhabra S., Hoimes C., Vogelzang N.J., Boyd T., Bergerot P.G., Ba J.J.A. (2019). A phase 1 trial of SGN-CD70A in patients with CD70-positive, metastatic renal cell carcinoma. Cancer.

[46] Allogene Therapeutics (2021). A Phase 1 Multicenter Study Evaluating the Safety and Efficacy of ALLO-316 Following ALLO-647 Containing Conditioning Regimen in Subjects with Advanced or Metastatic Clear Cell Renal Cell Carcinoma. clinicaltri-als.gov. https://clinicaltrials.gov/ct2/show/NCT04696731.

[47] Titov A., Zmievskaya E., Ganeeva I., Valiullina A., Petukhov A., Rakhmatullina A., Miftakhova R., Fainshtein M., Rizvanov A., Bulatov E. (2021). Adoptive Immunotherapy beyond CAR T-Cells. Cancers.

[48] Choueiri T.K., Plimack E.R., Bauer T.M., Merchan J.R., Papadopoulos K.P., McDermott D.F., Michaelson M.D., Appleman L.J., Thamake S., Zojwalla N.J. (2020). Phase I/II study of the oral HIF-2 α inhibitor MK-6482 in patients with advanced clear cell renal cell carcinoma (RCC). J. Clin. Oncol..

[49] Merck Sharp & Dohme Corp An Open-Label, Randomized, Phase 3 Study of MK-6482 in Combination with Lenvatinib (MK-7902) vs. Cabozantinib for Second-Line or Third-Line Treatment in Participants with Advanced Renal Cell Carcinoma Who Have Progressed after Prior Anti-PD-1/L1 Therapy. https://clinicaltrials.gov/ct2/show/NCT04586231.

[50] ClinicalTrials.gov A Study of Atezolizumab in Combination with Cabozantinib Compared to Cabozantinib Alone in Participants with Advanced Renal Cell Carcinoma after Immune Checkpoint Inhibitor Treatment (CONTACT-03). https://www.clinicaltrials.gov/ct2/show/NCT04338269.

[51] Agarwal N., Vaishampayan U., Green M., Di Nucci F., Chang P.-Y., Scheffold C., Pal S. (2018). Phase IB study (COSMIC-021) of cabozantinib in combination with atezolizumab: Results of the dose escalation stage in patients (pts) with treatment-naïve advanced renal cell carcinoma (RCC). Ann. Oncol..

[52] Kwilas A.R., Ardiani A., Donahue R.N., Aftab D., Hodge J.W. (2014). Effects of Cabozantinib, a small molecule tyrosine kinase inhibitor, on the immune permissiveness of the tumor microenvironment and immune-mediated killing of tumor cells. J. Immunother. Cancer.

